# The role of LEAP2 on cognitive impulsivity after refeeding: evidence from a preclinical study in female mice and from patients with anorexia nervosa

**DOI:** 10.1192/j.eurpsy.2026.10228

**Published:** 2026-07-31

**Authors:** C. Tezenas Du Montcel, H. H. Hamelin, N. Lebrun, N. Ramoz, P. Duriez, P. Gorwood, O. Viltart, V. Tolle

**Affiliations:** 1 Institute of Psychiatry and Neuroscience of Paris (IPNP), INSERM U1266, Université Paris Cité; 2 Hôpital Sainte Anne (CMME), GHU-Paris Psychiatrie et Neurosciences, Paris, France; 3 Mental Health Institute of the Douglas Hospital, McGill University, Montrel, Canada; 4 SCALab - Sciences Cognitives et Sciences Affectives, CNRS UMR 9193, PsySEF Faculty, Université de Lille, Villeneuve d’Ascq, France

## Abstract

**Introduction:**

Cognitive impulsivity is referred as the increased preference for an immediate rather than a delayed reward, evaluated in a delay discounting task (DDT) in both humans and rodents. Patients with anorexia nervosa (AN) exhibit an increased sensitivity for delayed reward particularly in acute stages of the disorder (Schuman et *al.* Sci. Rep. 2025;). Recent findings suggest that the ghrelin/LEAP2 (Liver-Expressed Antimicrobial Peptide 2) ratio impacts the dynamics of reward sensitivity (Andreoli et *al.* Diab Obes. & Metab 2024), and that LEAP2 could potentially serve as a biomarker of remission in patients with AN (Tezenas du Montcel *et al.* iScience 2023). We hypothesized that the ghrelin/LEAP2 balance was involved in impulsive food choices during refeeding processes following prolonged food restriction.

**Objectives:**

We investigated how changes in cognitive impulsivity correlate with the ghrelin/LEAP-2 balance during food restriction and following weight restoration through a translational study in mice and patients with AN.

**Methods:**

Impulse control and plasma ghrelin and LEAP2 concentrations were evaluated in a longitudinal study of 30 female patients with AN in the acute stage of the disorder, after weight restoration and 6-months following hospital discharge. Cognitive impulsivity was also assessed in young C57Bl6/J female mice at baseline, after a 15-day 50% quantitative food restriction and following a 10-day refeeding (n=8-12/group). The DDT task was performed in operant conditioning chambers as the choice for a small-immediate or a large-delayed delayed delivered with increasing delays (0 to 40 seconds). We collected blood for ghrelin and LEAP2 measurement and brain areas involved in metabolic response or reward and cognitive control.

**Results:**

Ghrelin/LEAP2 ratio was negatively correlated with impulse control in patients after weight restoration only in patients maintaining weight gain after discharge (n=14) but not in patients with unstable remission (n=16). In mice, food restriction increased cognitive impulsivity and refeeding only partially restored this phenotype compared to control conditions. Cognitive impulsivity was also positively correlated with plasma LEAP2 levels but not with gene expression of main hypothalamic neuropeptides or dopamine D1/D2 receptors in mesocorticolimbic structures.

**Conclusions:**

Our results suggest that the interaction between LEAP2 and cognitive impulsivity is affected by changes in nutritional status in patients and female mice. A prolonged episode of food restriction enhances cognitive impulsivity even after weight regain, which is correlated with plasma LEAP2 concentrations. Metabolic and cognitive consequences of food restriction could be implicated in how food choices are being modified in patients and be associated with a greater likelihood of achieving stable remission in AN.

**Disclosure of Interest:**

C. Tezenas Du Montcel: None Declared, H. Hamelin: None Declared, N. Lebrun: None Declared, N. Ramoz: None Declared, P. Duriez: None Declared, P. Gorwood Consultant of: P. G. received during the last 5 years fees for presentations at congresses or participation in scientific boards from Angelini, Biogen, Janssen, Lundbeck, Merk, Newron, Otsuka, Richter and Viatris, O. Viltart: None Declared, V. Tolle: None Declared

